# Acute Calculous Cholecystitis: What is new in diagnosis and therapy?

**DOI:** 10.1155/1992/46529

**Published:** 1992

**Authors:** Dirk J. Gouma, Huug Obertop

**Affiliations:** 1 Departments of Surgery University Hospital Maastricht The Netherlands; 2 Departments of Surgery University Hospital Utrecht The Netherlands

## Abstract

The management of patients with acute calculous cholecystitis has changed during recent years. The etiology of acute cholecystitis is still not fully understood. Infection of bile is relatively unimportant since bile and gallbladder wall cultures are sterile in many patients with acute cholecystitis. Ultrasonography is first choice for diagnosis of acute cholecystitis and cholescintigraphy is second best. Percutaneous puncture of the gallbladder that can be used for therapeutic drainage has also diagnostic qualities. Early cholecystectomy under antibiotic prophylaxis is the treatment of choice, and has been shown to be superior to delayed surgery in several prospective trials. Mortality can be as low as 0.5% in patients younger than 70–80 years of age, but a high mortality has been reported in octogenerians. Selective intraoperative cholangiography is now generally accepted and no advantage of routine cholangiography was shown in clinical trials. Percutaneous cholecystostomy can be successfully performed under ultrasound guidance and has a place in the treatment of severely ill patients with acute cholecystitis. Laparoscopic cholecystectomy can be done safely in patients with acute cholecystitis, but extensive experience with this technique is necessary. Endoscopic retrograde drainage of the gallbladder by introduction of a catheter in the cystic duct is feasible but data are still scarce.


*HPB Surgery*, 1992, Vol. 6, pp. 69–78
Reprints available directly from the publisher
Photocopying permitted by license only

© Harwood Academic Publishers GmbH
Printed in the United States of America

### REVIEW ARTICLE

### ACUTE CALCULOUS CHOLECYSTITIS

### What is new in diagnosis and therapy?

DIRK J. GOUMA* and HUUG OBERTOP**
*Departments of Surgery, University Hospital Maastricht* and Utrecht***

*(Received 2 April 1992)*

The management of patients with acute calculous cholecystitis has changed during recent years. The etiology of acute cholecystitis is still not fully understood. Infection of bile is relatively unimportant since bile and gallbladder wall cultures are sterile in many patients with acute cholecystitis. Ultrasonography is first choice for diagnosis of acute cholecystitis and cholescintigraphy is second best. Percutaneous puncture of the gallbladder that can be used for therapeutic drainage has also diagnostic qualities. Early cholecystectomy under antibiotic prophylaxis is the treatment of choice, and has been shown to be superior to delayed surgery in several prospective trials. Mortality can be as low as 0.5% in patients younger than 70–80 years of age, but a high mortality has been reported in octogenerians. Selective intraoperative cholangiography is now generally accepted and no advantage of routine cholangiography was shown in clinical trials. Percutaneous cholecystostomy can be successfully performed under ultrasound guidance and has a place in the treatment of severely ill patients with acute cholecystitis. Laparoscopic cholecystectomy can be done safely in patients with acute cholecystitis, but extensive experience with this technique is necessary. Endoscopic retrograde drainage of the gallbladder by introduction of a catheter in the cystic duct is feasible but data are still scarce.

KEY WORDS: Cholecystitis, gallstones

### INTRODUCTION

As a result of the development of new treatment modalities for gallstone disease such as ESWL, laparoscopic cholecystectomy and percutaneous cholecystostomy the management of patients with symptomatic gallstones has changed during recent years[1–3]. Cholecystectomy is still the procedure of choice for the six to twenty percent of these patients with acute cholecystitis[4–6]. Some aspects of the management of these patients are controversial. The aim of this review is to analyse changes in the diagnostic and therapeutic procedures for acute cholecystitis.

### *Etiology*

Acute cholecystitis is caused by gallstones in about 90–95% of patients[2,4,6,7]. The remaining 5–10% of patients suffer from acute acalculous cholecystitis, most frequently after surgery or during treatment in an intensive care unit. Acalculous disease of the gallbladder has been reviewed recently and will not be included in this review[7,8]. It is generally thought that gallstones cause obstruction of the cystic duct leading to changes of the bile flow giving rise to an inflammatory process within the gallbladder wall. Although the pathogenesis is not completely understood acute obstruction and stasis of bile are almost certainly both contributing factors. The infection of bile is relatively less important in this early stage since bile or gallbladder wall cultures are sterile in about 30–60% of the patients with acute cholecystitis[9–11].

Other factors such as prostaglandins as a mediator for inflammation or delayed gallbladder emptying in diabetic patients may also play a role in the development of acute cholecystitis but the mechanism is not yet fully understood[12–14].

### *Signs and Symptoms*

The clinical features of acute (calculous) cholecystitis have not changed over the years. Patients usually present with pain in the upper right quadrant of the abdomen. The pain is persistent and frequently radiates to the epigastrium or to the back and cannot easily be differentiated from biliary colic especially at the beginning of the disease. Kune describes: "the illness begins with an attack of biliary colic type of pain, but the pain does not settle down and remains unabated for one or more days"[15]. Nausea and vomiting are found as frequently as 86%. Mild fever (up to 38°C) is present in about half of the patients[16].

At physical examination right upper quadrant tenderness, localised peritonitis and a positive Murphy's sign are found in the majority of patients. Murphy's sign is elicited according to Bailey and Love's *Short practice of surgery*: " . . . by asking the patient to breathe in whilst gently palpating the gallbladder area. The patient will experience pain and 'catch her breath' just before the zenith of inspiration"[17]. Generalised peritonitis is rare (in our series only 7%) and highly suspicious of perforation of the gallbladder[16]. A palpable mass is present in about one third of the patients. Laboratory examination is not specific for the diagnosis[3,4,16]. White blood cell count can be elevated up to 15 × $10^9$/L and a mild elevation of liver function tests and serum bilirubin can be found, that is probably due to inflammation around the gallbladder and Calot's Triangle. Also associated common bile duct stones that are present in approximately 20% of the patients and more frequently in the elderly can give rise to biliary obstruction and elevated liver function tests and serum bilirubin[18–19].

### *Diagnostic Procedures*

Although a *plain abdominal roentgenogram* will be used frequently in patients with acute abdominal pain, the study is of limited value for the diagnosis of acute cholecystitis[21,22]. Only calcified stones, that are present in about 20% of patients with cholelithiasis, can be visualised. The study is however highly sensitive when air bubbles in the lumen or wall of the gallbladder are seen, caused by gas forming organisms such as *E. coli* and *Clostrida.*

*Ultrasonography* is nowadays the most commonly used test for screening of patients with acute cholecystitis. It is relatively easy to perform, available at many locations (such as intensive care unit) and non-invasive. The sensitivity and specificity of the test for acute cholecystitis is respectively 90–95% and 70–98% depending whether so called major or minor criteria are being used[23–26]. Major criteria are: gallstones and non-visualisation of the gallbladder. Minor criteria are: thickening of the gallbladder wall of more than 5 mm, tenderness of the gallbladder when palpated during the examination, gallbladder enlargement to more than 5 cm, a round gallbladder shape and pericholecystic fluid. Controversy exists over minor criteria: tenderness and pericholecystic fluid, since these can be considered major criteria because of the high specificity for acute cholecystitis. Ultrasonography is also useful in detecting other, non-gallbladder related causes of abdominal pain.

*Cholescintigraphy* is being used increasingly during the last 10 years, after the development of newer and better radioisotopic labelled substances[23,25,27–30]. These substances are excreted by the liver into the bile, thus visualising bile flow into the intestinal tract and also through the cystic duct into the gallbladder. For patients with acute cholecystitis non-visualisation of the gallbladder has to be related to visualisation of the intestinal tract. Non-visualisation of both gallbladder and intestinal tract implies delayed hepatic clearance or biliary obstruction.

Pericholecystic hepatic uptake has been described as a valuable secondary sign in the cholescintigraphic diagnosis of acute cholecystitis[30].

Both the sensitivity and specificity of the tests (with different substances) vary between 90–97%[23,25,27–30]. Administration of low dose morphine especially in critically ill patients can reduce the number of false positive tests[31,32]. However the number of false positive tests is high in patients with hepatitis, pancreatitis, in patients receiving parenteral nutrition and in alcoholics.

A *percutaneous puncture* of the gallbladder that can be used for therapeutic drainage has also important diagnostic qualities[33,34]. This feature will be dealt with later, when discussing percutaneous cholecystostomy, a technique used in critically ill patients[35]. Since the puncture can be performed under ultrasound guidance without moving the patient to another unit, it has advantages above the more cumbersome cholescintigraphy.

*Computer tomography* (CT) has been reported to be successful in patients with acalculous cholecystitis but this modality is not used routinely for acute cholecystitis[25].

*Magnetic resonance imaging* (MRI) is not yet fully evaluated but its role in acute cholecystitis so far seems limited.

*Oral cholecystogram* is not used because it will take 14–18 hours before optimal opacification the gallbladder and because of the sensitivity for acute cholecystitis is low[23,36].

*Intravenous cholangiography* (IVC) has been used frequently in the past but visualisation of the biliary tract was only limited. Therefore the method is seldomly used since the introduction of ultrasonography and cholescintigraphy. An IVC can sometimes be useful when the diagnosis is uncertain. Opacification of the gallbladder makes acute cholecystitis unlikely[26].

In summary ultrasonography is first choice for diagnosis of acute cholecystitis and cholescintigraphy is second best. The last method is used less frequently nowadays in severely ill patients since percutaneous ultrasound-guided punctures are favoured for diagnostic and therapeutic purposes.

### *Treatment*

After acute cholecystitis has been diagnosed, appropriate treatment and timing of this treatment has to be selected for the individual patient. Surgery is still the treatment of choice for acute cholecystitis. Use and type of antibiotics and timing and type of surgery will be discussed.

*Use of antibiotics*

Broad spectrum antibiotics are usually thought to be indicated as prophylaxis in the perioperative period[16]. However, opinions on the specific antibiotic regimen may differ. Indeed acute cholecystitis is not an infectious disease, since in 30–60% of the patients no bacteria can be shown in the bile[9–11]. On the other hand acute cholecystitis is considered as one of the factors (together with high age, diabetes mellitus, common bile duct stones, previous biliary surgery) known to be associated with an increased risk of positive cultures[13,19,37]. The highest incidence of bactibilia is found within 24 hours after onset of acute cholecystitis[10]. Postoperative infections have been shown to occur in about 20% of patients with bactibilia and in 2.5% of the patients with sterile bile[37]. Unfortunately bile cultures can only be known after surgery, whereas the choice for antibiotics has to be made before operation. Elevation of temperature (> 37.3°C), serum bilirubin level (> 8.6 mol/L) and white blood cell count (> $14.10^9$/L) have been shown to predict bactibilia in patients with acute cholecystitis[38]. It has been suggested that patients with 0–1 of these factors receive one single (preoperative) dose whereas in patients with 2–3 factors antibiotics are continued until the outcome of a bile culture is available[38]. In one meta-analysis of antibiotic prophylaxis in biliary surgery the overall reduction of wound infection was 9%[39]. The reduction was significantly greater (13% and 25% respectively for early and late wound infection) for high risk patients including patients with acute cholecystitis. No difference between single and multiple dose regimens could be found, but in most trials studying different dosages different antibiotics were also used[39]. The choice of an antibiotic should be based on the organisms most commonly found in bile, such as E. coli, Klebsiella, Enterobacter, Proteus, Streptococcus and Staphylococcus faecalis. The antibiotic should be present in the tissue during the time of contamination. It is still not clear whether those antibiotics that reach a high concentration in the biliary tract should be chosen since the cystic duct is obstructed in acute cholecystitis[39–41]. Even in a prospective study in patients with acute cholangitis using Mezlocillin with a high biliary excretion no advantage over treatment with ampicillin and tobramycin having a lower biliary excretion was found[42,43]. Cephalosporins or a combination of penicillins with gentamycin or metronidazole is the most commonly used prophylactic regimen in the Netherlands[44].

Generally patients with acute cholecystitis are not being treated with antibiotics for a long period of time as primary treatment and surgery or biliary drainage is the treatment of choice[43]. However in those few patients not being operated on or treated by drainage, antibiotics can be used in order to prevent complications of the disease caused by bactibilia[45]. The choice of the antibiotics in these circumstances will be the same as selected for prophylaxis and should be continued for 5–7 days[39,44]. The difference between acute cholecystitis and acute cholangitis can be difficult especially in jaundiced patients[18,19]. For these patients treatment with antibiotics is mandatory although biliary drainage should be instituted without much delay.

*Cholecystectomy and timing of surgery*

There is agreement in the literature that cholecystectomy is the procedure of choice for most patients with acute cholecystitis[1,40]. It has been discussed for many years whether surgery should be performed early (within 24–48 hours after admission) or delayed (after 2–3 months). The results of randomized trials are strongly in favour of early cholecystectomy[47–51], showing no difference in post operative mortality (0–1%) and morbidity for the two strategies but a clear reduction in hospital stay[47–51]. In one paper the mean total hospital stay was 10.1 days in the early and 18.9 days in the delayed surgery group. When delayed surgery was chosen 10–20% of the patients had to be operated earlier than planned because of a downhill clinical course[52].

Despite the outcome of these randomized trials many surgeons still favour delayed operation, especially when patients are admitted more than 48 hours after the onset of symptoms. This hesitation for cholecystectomy after a somewhat longer duration of symptoms probably reflects the philosophy that operating in severely edematous and inflamed tissue in Calot's Triangle carries an elevated operative risk. The influence of duration of the onset of symptoms and results of surgery was recently studied[16]. No difference was found in mortality and morbidity with regards to duration of symptoms (<2 days versus 2–7 days). The overall mortality was 1.6% (2 patients of 88 and 89 years died postoperatively). Early cholecystectomy is a safe procedure for patients with acute cholecystitis at least within 7 days after onset of symptoms[16]. Other studies of the last ten years have shown a gradual improvement of the results of surgery for acute cholecystitis, mortality being less than 0.5% in patients younger than 70–80 years of age[16,53–56]. However the operative risk can be considerable in patients over 74 years of age[57] and a mortality as high as 11.6% has been reported after biliary surgery in octogenerians[58]. Perforation of the gallbladder because of acute cholecystitis still carries a high mortality. Especially in this group of patients early surgery is mandatory[59,60].

*Intraoperative cholangiography*

The efficacy of routine intraoperative cholangiography in patients that undergo elective surgery for symptomatic gallstone disease has frequently been challenged[61–64]. Selective cholangiography has been accepted generally in Europe now since no advantage of routine cholangiography was shown in clinical trials[61–64]. However especially in elderly patients with acute cholecystitis the incidence of common bile duct stones is as high as 50% and therefore routine cholangiography can be possibly recommended in this group. Cholangiography can also visualise the anatomy of the biliary tract and surgical trauma can so be prevented. On the other hand cholangiography is more difficult in patients when severe inflammation is present in Calot's Triangle and the obstruction of the cystic duct makes easy access for cholangiography impossible. Therefore we advise performing cholangiography selectively when it is possible without additional risk. When cholangiography cannot be performed safely ERCP and papillotomy may be necessary shortly after operation to provide adequate biliary drainage. But when the suspicion of common bile duct stones is very high (in elderly patients with jaundice or a dilated biliary tree on ultrasound) one should consider cholangitis as the main cause of the symptoms and ERCP and papillotomy should be performed as the first treatment and cholecystectomy can follow later if still indicated[65–67].

*Open or percutaneous cholecystostomy*

Cholecystostomy can be used in the treatment of acute cholecystitis for various indications some of which are obsolete, some still valid. Cholecystostomy can be a very prudent procedure: Firstly when morbidity and mortality of cholecystectomy under general anaesthesia is high. In these patients cholecystostomy could be performed under local anaesthesia[35,68–71]. Secondly, when technical difficulties are expected in operations in a severely inflamed Calot's Triangle. And thirdly and more currently the percutaneous approach is favoured for critically ill patients in an intensive care unit for diagnosis and treatment of acute (acalculous) cholecystitis[33,34]. Cholecystostomy can be performed as a definitive procedure for calculous cholecystitis[72–73]. With ultrasound guidance an incision is made directly over the fundus of the gallbladder and after a purse string suture, bile is aspirated, stones are removed and a catheter is left behind. Tube cholangiography can be performed postoperatively in these patients and retained stones can so be removed or dissolved by MTBE and eventually "sclerosing" of the gallbladder mucosa can be performed[72–76]. Elective cholecystectomy can also be considered after improvement of the patients general condition. The risk of cholecystostomy mainly depends on the clinical condition of the patient. Frequently old studies are quoted. Mortality of open cholecystostomy for acute cholecystitis varies between 4 and 36% and morbidity between 10–60%[72,71,74,77]. About 50% of patients will develop symptomatic biliary tract disease two years after cholecystostomy. Others have reported that the need for a cholecystectomy at a later date is only exceptionally indicated and that more than 90% of the patients are asymptomatic after 1–12 years. The advantage of the open procedure over the percutaneous technique is that necrosis and perforation of the gallbladder that are more common in elderly, diabetic patients can be treated using the former technique[71,72]. The risk of peritoneal leak seems to be minimal although data about this complication after both methods are rather confusing.

A subtotal cholecystectomy as advocated by Terblanche can be considered as a minimal modification of the open cholecystostomy[78].

Percutaneous cholecystostomy under ultrasound guidance is relatively easy to perform in severely ill patients in an intensive care unit. The diagnostic accuracy of the method is high as is the success rate of puncture as therapy. Morbidity and mortality are reported respectively 25% and 10% being very much dependent on patient selection. The procedure can also be followed by cholangiography and cholecystolithotomy or dissolution by MTBE[75]. The percutaneous approach to the gallbladder can be performed by a direct puncture or via the transhepatic route. The first technique is only possible in a limited number of patients because of the localisation of either liver or colon. The latter is nearly always possible but coagulation disorders should be corrected preoperatively. The possibility of not detecting necrosis and perforation of the gallbladder is already mentioned earlier.

### *Other Minimally Invasive Techniques*

Laparoscopic cholecystectomy has been largely restricted to patients with symptomatic gallstone disease[79–81]. Acute cholecystitis is considered by most surgeons as a contraindication for this procedure. However when a large experience of this new technique has been obtained laparascopic cholecystectomy can be used in patients with acute cholecystitis. In the first consecutive series of patients with acute cholecystitis treated by this technique a success rate of 66% (10/15) was reported without complications[82]. Five patients underwent laparotomy. The mean hospital stay was 2.7 days and the length of the operative procedure was 126 minutes as compared with around 90 minutes for laparoscopic cholecystectomy in non-acute cholecystitis. Cholangiography could be performed in 14/15 patients. These preliminary results suggest that laparoscopic cholecystectomy can be done safely in patients with acute cholecystitis. However, the authors have stressed that this procedure should only be attempted after extensive experience with laparoscopic biliary tract surgery has been obtained. A low threshold to perform laparotomy is advised.

### *Endoscopic Retrograde Cholecysto-endoprosthesis*

Recently endoscopic placement of an endoprosthesis into the cystic duct has been performed in 14 patients with (sub)acute cholecystitis[83]. This new technique was studied in patients with abdominal pain, leukocytosis, thickening of the gallbladder wall and fever. However patients "in need for surgery" were excluded, although the criteria were not clearly stated. Clinical improvement was found in two third of patients. These early results only show that successful introduction of a catheter in the cystic duct is possible but more data and comparison with surgical techniques have to be awaited.

### *Summary*

Although acute calculous cholecystitis is a clear and unchanged clinical entity, the diagnostic and therapeutic procedures have been changed during the past years. Ultrasound is first choice for the diagnosis and cholescintigraphy is second best. However the percutaneous ultrasound guided puncture has become more popular having not only important diagnostic qualities but also therapeutic possibilities. The method is easy to perform in severely ill patients for instance in an intensive care unit and the diagnostic accuracy is high.

Preoperative antibiotics are mandatory in the treatment of acute cholecystitis (surgical as well as drainage). There is still debate on the choice of single or multi drug regimens, and type of antibiotics. Antibiotics are not a substitution for adequate drainage especially in jaundiced patients in which the differentiation between acute cholecystitis and cholangitis can sometimes be difficult.

Early cholecystectomy is the treatment of choice at least within 7 days after onset of symptoms. During surgery selective cholangiography is favoured by ourselves, whereas others still recommend routine cholangiography. There is not much need for this in difficult circumstances (severe inflammation) when ERCP and papillotomy is widely available.

For patients with high operative risk and critically ill patients percutaneous cholecystostomy is a good alternative treatment. The procedure can be followed by cholecystolithotomy or dissolution therapy.

Open cholecystostomy should probably be reserved when technical difficulties arise or are expected at cholecystectomy.

Laparoscopic cholecystectomy for acute cholecystitis is possible in some patients but should only be performed by surgeons with a large experience in laparoscopic surgery and a low threshold to convert into an open procedure is generally advised. Endoscopic cholecystic drainage is a new technique of which results should be awaited.

### *References*

1. Cheslyn-Curtis, S. and Russel, R.C.G. (1991) New trends in gallstone management. *Br. J. Surg.*, **78**, 143–149

2. Mack, E. (1990) Role of surgery in the management of gallstones. Seminars in liver disease. **10**, 222–231
3. Irvin, T.T. and Arnstein, P.M. (1988) Management of symptomatic gallstones in the elderly. *Br. J. Surg.*, **75**, 1163–1165
4. Hermann, R.E. (1990) Surgery for acute and chronic cholecystitis. *Surg. Clin. North Am.*, **70**, 1263–1275
5. van Rensburg, L.C.J. (1984) The management of acute cholecystitis in the elderly. *Br. J. Surg.*, **71**, 692–693
6. Sharp, K.W. (1988) Acute cholecystitis. *Surg. Clin. North Am.*, **68**, 269–279
7. Williamson, R.C.N. (1988) Acalculous disease of the gallbladder. *Gut*, **29**, 860–872
8. Goris, R.J.A. (1986) Acute acalculous cholecystitis. *Neth. J. Surg.*, **38**(4), 106–108
9. Claesson, B.E.B., Holmlund, D.E.W. and Mäzsch, T.W. (1984) Biliary microflora in acute cholecystitis and the clinical implications. *Acta Chir. Scand.*, **150**, 229–237
10. Claesson, B.E.B., Holmlund, D.E.W. and Mätsch, T.W. (1986) Microflora of the gallbladder related to duration of acute cholecystitis. *Surg. Gynecol. Obstet.*, **162**, 531–535
11. Järvinen, H.J. (1980) Biliary bacteremia at various stages of acute cholecystitis. *Acta Chir. Scand.*, **146**, 427–430
12. Ikard, R.W. (1990) Gallstones, cholecystitis and diabetes. *Surg. Gynecol. Obstet.*, **171**, 528–531
13. Hickman, M.S., Schwesinger, W.H. and Page, C.P. (1988) Acute cholecystitis in the diabetic. *Arch. Surg.*, **123**, 409–411
14. Kaminsky, D.L., Andrus, C.H., German, D. and Desphande, Y.G. (1990) The role of prostanoids in the production of acute acalculous cholecystitis by platelet-activating factor. *Ann. Surg.*, **212**(4), 455–461
15. Kune, J.A. and Sali, A. (1980) *The practice of biliary surgery*, 2 edt. Oxford: Blackwell
16. Hoppenbrouwer, F.H. and Gouma, D.J. (1990) Early cholecystectomy for acute cholecystitis; effect of length of symptoms on morbidity and mortality. *Ned. Tijdschr. Geneeskd.*, **134**(47), 2293–2296
17. Bailey and Love (1992) *Short practice of surgery*, 21st edition. London: Chapman and Hall
18. Coelho, J.C.U., Buffara, M., Pozzobon, C.E. *et al.* (1984) Incidence of common bile duct stones in patients with acute and chronic cholecystitis. *Surg. Gynecol. Obstet.*, **158**, 76–80
19. Glenn, F. and Dillon, L.D. (1980) Developing trends in acute cholecystitis and choledocholithiasis. *Surg. Gynecol. Obstet.*, **151**, 528–532
20. Houghton, P.W.J., Jenkinson, L.R. and Donaldson, L.A. (1985) Cholecystectomy in the elderly: a prospective study. *Br. J. Surg.*, **72**, 220–222
21. Eisenberg, R.L., Heineken, P., Hedgcock, M.W. *et al.* (1982) Evaluation of plain abdominal radiographics in the diagnosis of abdominal pain. *Ann. Intern. Med.*, **97**, 257–261
22. Rosenquist, C.J. (1981) Radiology of the biliary tree. *Surg. Clin. North. Am.*, **61**, 775–786
23. Marton, K.I., Doubilet, P. (1988) How to image the gallbladder in suspected cholecystitis. *Ann. Intern. Med.*, **109**, 722–729
24. de Lacey, G., Twomey, B., Gajjar, B. *et al.* (1984) Should cholecystography or ultrasound be the primary investigation for gallbladder disease? *Lancet*, **I**, 205–207
25. Mirvis, S.E., Vainright, J.R., Nelson, A.W. *et al.* (1986) The diagnosis of acute acalculous cholecystitis: a comparison of sonography, scintigraphy, and CT. *AJR*, **147**, 1171–1175
26. Norrby, S., Frank, M. and Sjödahl, R. (1985) Intravenous cholecystography and ultrasonography in the diagnosis of acute cholecystitis. *Acta Chir. Scand.*, **151**, 255–259
27. Fink-Bennett, D., Freitas, J.E., Ripley, S.D. *et al.* (1985) The sensitivity of hepatobiliary imaging and real-time ultrasonography in the detection of acute cholecystitis. *Arch. Surg.*, **120**, 904–906
28. Gill, P.T., Dillon, E., Leahy, A.L. *et al.* (1985) Ultrasonography, HIDA scintigraphy or both in the diagnosis of acute cholecystitis? *Br. J. Surg.*, **72**, 267–268
29. Garner, W.L., Marx, V. and Fabri, P.J. (1988) Cholescintigraphy in the critically ill. *Am. J. Surg.*, **155**, 727–729
30. Swayne, L.C. and Ginsberg, H.N. (1989) Diagnosis of acute cholecystitis by cholescintigraphy: significance of pericholecystic hepatic uptake. *AJR*, **152**, 1211–1213
31. Kim, E.E., Pjura, G., Lowry, P. *et al.* (1986) Morphine-augmented cholescintigraphy in the diagnosis of acute cholecystitis. *AJR.*, **147**, 1177–1179
32. Flancbaum, L., Alden, S.M. and Trooskin, S.Z. (1989) Use of cholescintigraphy with morphine in critically ill patients with suspected cholecystitis. *Surgery*, **106**, 668–674
33. Eggermont, A.M., Laméris, J.S. and Jeekel, J. (1985) Ultrasound-guided percutaneous transhepatic cholecystostomy for acute acalculous cholecystitis. *Arch. Surg.*, **120**, 1354–1356

34. McGahan, J.P. and Lindfors, K.K. (1988) Acute cholecystitis: diagnostic accuracy of percutaneous aspiration of the gallbladder. *Radiology*, **167**, 669–671
35. Lee, M.J., Saini, S., Brink, J.A. *et al.* (1991) Treatment of critically ill patients with sepsis of unknown cause: value of percutaneous cholecystostomy. *AJR.*, **156**, 1163–1166
36. Burhenne, H.J. and Obata, W.G. (1975) Single-visit oral cholecystography. *N. Engl. J. Med.*, **292**, 627–628
37. Wells, G.R., Taylor, E.W., Lindsay, G. *et al.* (1989) Relationship between bile colonization, high risk factors and postoperative sepsis in patients undergoing biliary tract operations while receiving a prophylactic antibiotic. *Br. J. Surg.*, **76**, 374–377
38. Thompson, J.E., Benion, R.S., Doty, E. *et al.* (1990) Predictive factors for bactibilia in acute cholecystitis. *Arch. Surg.*, **125**, 261–264
39. Meijer, W.S., Schmitz, P.I.M. and Jeekel, J. (1990) Meta-analysis of randomized, controlled clinical trials of antibiotic prophylaxis in biliary tract surgery. *Br. J. Surg.*, **77**, 283–290
40. Aloj, G., Bianco, C., Covelli, I. *et al.* (1991) Antibiotic prophylaxis for biliary tract surgery: selection of patient and agent. *Int. Surg.*, **76**, 131–134
41. Editorial (1989) Antibiotics for cholangitis. *Lancet*, **2**, 781–782
42. Gerecht, W.B., Henry, N.K., Hoffman, W.W. *et al.* (1989) Prospective randomized comparison of mezlocillin therapy alone with combined ampicillin and gentamicin therapy for patients with cholangitis. *Arch. Intern. Med.*, **149**, 1279–1284
43. Thompson, J.E., Pitt, H.A., Doty, J.E. *et al.* (1990) Broad spectrum penicillin as an adequate therapy for acute cholangitis. *Surg. Gynecol. Obstet.*, **171**, 275–282
44. Meijer, W.S. (1990) Antibiotic prophylaxis in biliary tract surgery — Current practice in The Netherlands. *Neth. J. Surg.*, **42**(4), 96–100
45. Audisio, R.A., Bozzetti, F., Severini, A. *et al.* (1988) The occurrence of cholangitis after percutaneous biliary drainage: Evaluation of some risk factors. *Surgery*, **103**, 507–512
46. McSherry, C.K. (1989) Cholecystectomy: The gold standard. *Am. J. Surg.*, **158**, 174–178
47. van der Linden, W. and Sunzel, H. (1970) Early versus delayed operation for acute cholecystitis. A controlled clinical trial. *Am. J. Surg.*, **120**, 7–13
48. Lahtinen, J., Alhava, E.M. and Aukee, S. (1978) Acute cholecystis treated by early and delayed surgery. A controlled clinical trial. *Scand. J. Gastroentr.*, **13**, 673–678
49. van der Linden, W. and Edlund, G. (1981) Early versus delayed cholecystectomy: the effect of a change in management. *Br. J. Surg.*, **68**, 753–757
50. Norrby, S., Herlin, P., Holmin, T. *et al.* (1983) Early or delayed cholecystectomy in acute cholecystitis. A clinical trial. *Br. J. Surg.*, **70**, 163–165
51. McCarthur, P., Cuschieri, A., Sells, R.A. *et al.* (1975) Controlled clinical trial comparing early with interval cholecystectomy for acute cholecystitis. *Br. J. Surg.*, **62**, 850–852
52. Järvinen, H., Hästbacka, J., Turunen, M.I. (1979) The treatment of acute cholecystitis. *Acta Chir. Scand.*, **145**, 399--404
53. Edlund, G. and Ljungdahl, M. (1990) Acute cholecystitis in the elderly. *Am. J. Surg.*, **159**, 414–416
54. Addisson, N.V. and Finan, P.J. (1988) Urgent and early cholecystectomy for acute gallbladder disease. *Br. J. Surg.*, **75**, 141–143
55. Reiss, R., Nudelman, I., Gutman, C. *et al.* (1990) Changing trends in surgery for acute cholecystitis. *World J. Surg.*, **14**, 567–571
56. Hidalgo, L.A., Capellá, G. and Pi-Figueras, J. *et al.* (1989) The influence of age on early surgical treatment of acute cholecystitis. *Surg. Gynecol. Obstet.*, **169**, 393–396
57. van der Ham, A.C., Lange, J.F. and Yo, T.I. (1986) Acute cholecystitis in the elderly — A retrospective study. *Neth. J. Surg.*, **38**, 142–146
58. Landau, O., Deutsch, A.A., Nudelman, I. and Reiss, R. (1992) Surgery for acute cholecystitis in octogenarians. *HPB Surg.*, in press
59. Andersson, R., Tranberg, K.G. and Bengmark, S. (1990) Bile peritonitis in acute cholecystitis. *HPB Surg.*, **2**(1), 7–12
60. Larmi, T.K.I., Kairaluoma, M.I., Junila, J. *et al.* (1984) Perforation of the gallbladder. *Acta Chir. Scand.*, **150**, 557–560
61. Hauer-Jensen, M., Karesen, R., Nygaard, K. *et al.* (1986) Consequences of routine peroperative cholangiography during cholecystectomy for gallstone disease. *World J. Surg.*, **10**, 996–1002
62. Bogokowsky, H., Slutzki, S., Zaidenstein, L. *et al.* (1987) Selective operative cholangiography. *Surg. Gynecol. Obstet.*, **164**, 124–126

63. Gerber, A. (1986) A requiem for the routine operative cholangiogram. *Surg. Gynecol. Obstet.*, **163**, 363–364
64. Kitahama, A., Kerstein, M.D. and Overby, J.L. (1986) Routine intraoperative cholangiogram. *Surg. Gynecol. Obstet.*, **162**, 317–322
65. Lambert, M.E., Betts, C.D., Hill, J. *et al.* (1991) Endoscopic sphincterotomy: the whole truth. *Br. J. Surg.*, **78**, 473–476
66. Vaira, D., Ainley, C., Williams, S. *et al.* (1989) Endoscopic sphincterotomy in 1000 consecutive patients. *Lancet*, **ii**, 431–434
67. Siegel, J.H., Safrany, L., Ben-Zvi, J.S. *et al.* (1988) Duodenoscopic sphincterotomy in patients with gallbladder *in situ*: report of a series of 1272 patients. *Am. J. Gastroenterol.*, **83**, 1255–1258
68. Werbel, G.B., Nahrwold, D.L., Joehl, R.J. *et al.* (1989) Percutaneous cholecystostomy in the diagnosis and treatment of acute cholecystitis in the high-risk patient. *Arch. Surg.*, **124**, 782--786
69. Winkler, E., Kaplan, O., Gutman, M. *et al.* (1989) Role of cholecystostomy in the management of critically ill patients suffering from acute cholecystitis. *Br. J. Surg.*, **76**, 693–695
70. Klimberg, S., Hawkins, I. and Vogel, S.B. (1987) Percutaneous cholecystostomy for acute cholecystitis in high-risk patients. *Am. J. Surg.*, **153**, 125–129
71. McGahan, J.P. (1988) A new catheter design for percutaneous cholecystostomy. *Radiology*, **166**, 49–52
72. Moore, E.E., Kelly, G.L., Driver, T. *et al.* (1979) Reassessment of simple cholecystostomy. *Arch. Surg.*, **114**, 515--519
73. Kaufman, M., Weissberg, D. and Schwartz, I. (1990) Cholecystostomy as a definitive operation. *Surg. Gynecol. Obstet.*, **170**, 533–537
74. Griffith, D.P., Gleeson, M.J., Appel, M.F. *et al.* (1990) Percutaneous cholecystolithotomy. *Arch. Surg.*, **125**, 1114–1118
75. Malt, R., Hofmann, A., McSherry, C.K. *et al.* (1989) Panel discussion: Cholecystitis. *Am. J. Surg.*, **158**, 205–217
76. Burhenne, H.J. and Stoller, J.L. (1985) Minicholecystostomy and radiology stone extraction in high-risk cholelithiasis patients. *Am. J. Surg.*, **149**, 632–635
77. Skillings, J.C., Kumai, C. and Hinshaw, J.R. (1980) Cholecystostomy: a place in modern biliary surgery? *Am. J. Surg.*, **139**, 865–869
78. Bornman, P.C. and Terblanche, J. (1985) Subtotal cholecystectomy: for the difficult gallbladder in portal hypertension and cholecystitis. *Surgery*, **98**, 1–6
79. Meyers, W.C. (1991) A prospective analysis of 1518 laparoscopic cholecystectomies. *New Engl. J. Med.*, **324**, 1073–1078
80. Wastell, C. (1991) Laparoscopic cholecystectomy. Better for patients and the health service. *Br. Med. J.*, **303**, 303–304
81. Olsen, D.O. (1991) Laparoscopic cholecystectomy. *Am. J. Surg.*, **161**, 339–344
82. Flowers, J.L., Bailey, R.W., Scovill, W.A. *et al.* (1991) The Baltimore experience with laparoscopic management of acute cholecystitis. *Am. J. Surg.*, **161**, 388–392
83. Tamada, K., Seki, H., Sato, K. *et al.* (1991) Efficacy of endoscopic retrograde cholecystoendoprothesis (ERCCE) for cholecystitis. *Endoscopy*, **23**, 2–3

*(Accepted by S. Bengmark 3 February 1992)*

